# Case Report: Emergency management of difficult airway in a thyroid cancer patient with undiagnosed tracheal diverticulum preoperatively and literature review

**DOI:** 10.3389/fmed.2025.1739525

**Published:** 2026-01-02

**Authors:** Xianmei Chen, Chengyi Cai, Yangkun Li, Yong Wang

**Affiliations:** 1 The First Clinical Medical School of Guangzhou University of Chinese Medicine, Guangzhou, Guangdong, China; 2 Department of Anesthesiology, Maoming People’s Hospital, Maoming, Guangdong, China; 3 Department of Anesthesiology, The First Affiliated Hospital of Guangzhou University of Chinese Medicine, Guangzhou, Guangdong, China; 4 State Key Laboratory of Traditional Chinese Medicine Syndrome, The First Affiliated Hospital of Guangzhou University of Chinese Medicine, Guangzhou, Guangdong, China

**Keywords:** airway management, case report, fiberopticbronchoscopy, thyroid cancer, tracheal diverticulum

## Abstract

Tracheal diverticulum, a rare chronic airway pathology characterized by cystic outpouchings from the trachea or main bronchi, has scant epidemiological documentation. We present a thyroid cancer case with known airway stenosis but an undiagnosed tracheal diverticulum. Perioperatively, repeated displacement of the endotracheal tube into the undiagnosed diverticulum caused critical intubation failure and complicated tracheostomy. Under fiberoptic bronchoscopic guidance, the tracheal tube was successfully advanced past a right subglottic diverticular orifice into the left tracheal lumen, achieving secure placement in the true tracheal cavity. Our retrospective analysis of published cases further characterizes pathological features. For elective surgery patients with suspected tracheal diverticulum, particularly those with prior cervical surgery or pathology, we strongly advise preoperative bronchoscopy to confirm the defect and develop tailored airway management. This case also demonstrates that transnasal fiberoptic bronchoscope-guided intubation effectively rescues unanticipated difficult airways during tracheal diverticulum-related reintubation.

## 1 Introduction

Airway management in thyroid cancer patients presents substantial challenges, particularly when compounded by airway deformation, tracheal compression, and dyspnea (1). In such high-risk scenarios, conventional rapid-sequence induction risks triggering complete airway obstruction. For managing these complex airways, the 2022 American Society of Anesthesiologists Practice Guidelines for Management of the Difficult Airway (hereafter referred to as the 2022 ASA guidelines) specify: Awake tracheal intubation (ATI) is recommended for patients with predictors of difficult airway management or existing ventilation/oxygenation compromise (2). Furthermore, flexible bronchoscopy (FOB) is generally regarded as the preferred technique for anticipated difficult airways. However, the 2022 ASA guidelines lack explicit recommendations for managing unanticipated tracheal diverticula (TD) encountered during ATI.

TD is rare, with reported incidence rates of approximately 1% in adults and 0.3% in children (3). Most cases are asymptomatic and discovered incidentally through imaging, bronchoscopy, or biopsy, contributing to significant underdiagnosis. First reported by Rokitansky in 1938, TD was not formally characterized until 1954 when Mathey et al. established TD as a relatively benign and asymptomatic entity through pathologic examination of three cases (4). Anatomically, TD manifests as cystic lesions protruding beyond the tracheobronchial lumen. It can be classified into congenital and acquired types (5). Congenital TD typically presents with narrow necks, located 4 to 5 cm below the glottis or above the carina, and demonstrates normal tracheal wall architecture including smooth muscle, cartilage, and respiratory epithelium (6, 7). In contrast, acquired TD features wide-necked connections to the airway lumen, may occur at any tracheal level, and histologically lacks cartilaginous rings—consisting predominantly of respiratory epithelium (8–10).

We present an emergency difficult airway management case in a thyroid cancer patient with a preoperatively undiagnosed TD, admitted for endoscopic gastrostomy due to dysphagia. Given TD’s rarity and the consequent scarcity of documented clinical cases, we conducted a retrospective analysis of published cases to further investigate its characteristics.

## 2 Case description

**Figure 1:**
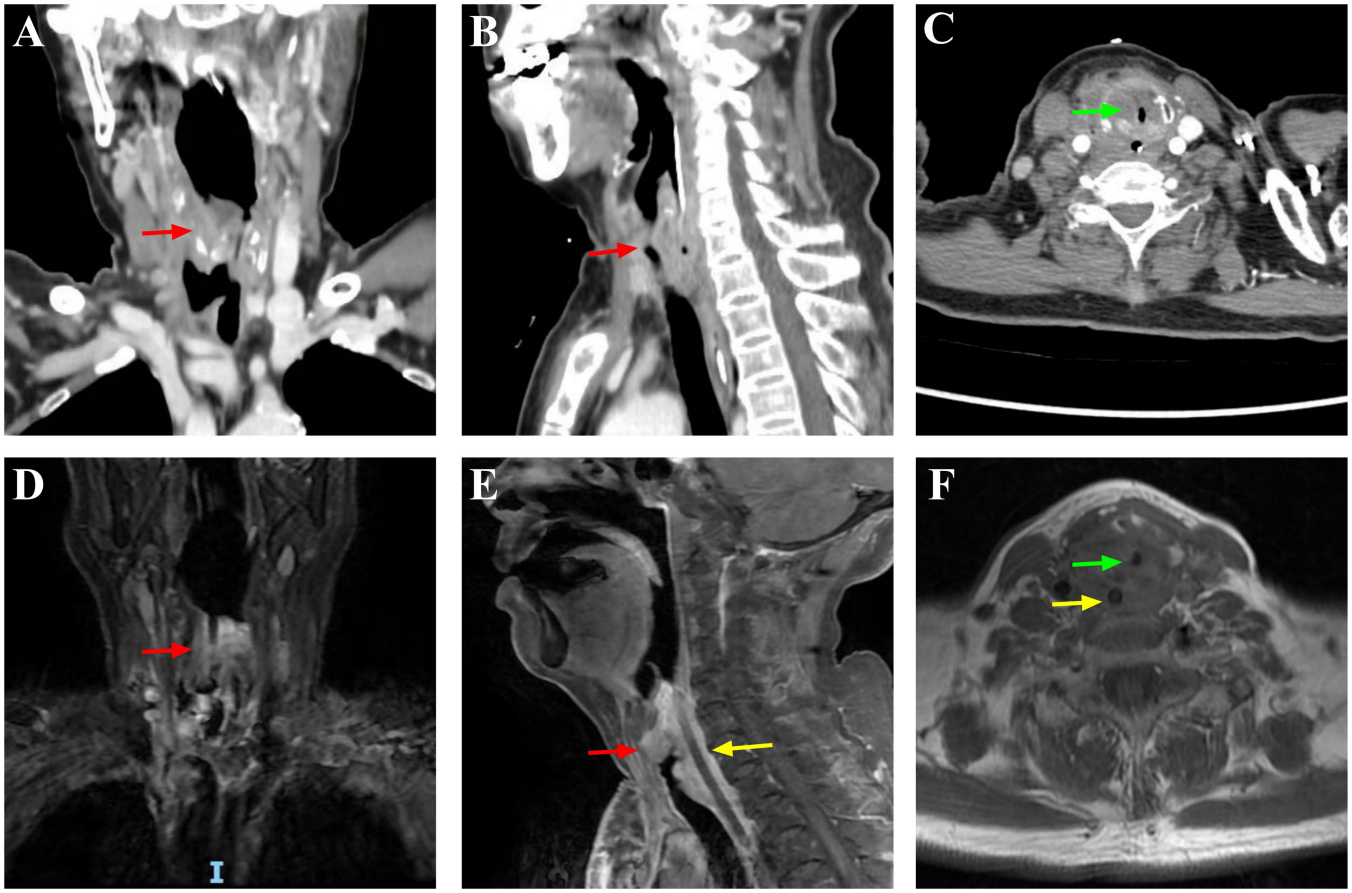
Preoperative neck imaging examination. CT: **(A)** Coronal view; **(B)** Sagittal view; **(C)** Axial view; MRI: **(D)** Coronal view; **(E)** Sagittal view; **(F)** Axial view. Red arrows in **(A,B,D,E)**: Irregular mass in the right thyroid lobe with indistinct borders and unclear demarcation from the adjacent trachea and esophagus; Yellow arrows in **(E,F)**: Tumor invasion into the esophagus; Green arrows in **(C,F)**: Subglottic laryngeal stenosis and tracheal lumen invasion/narrowing.

A 66-year-old woman, 156 cm in height and 46 kg in weight, was hospitalized for 2 months due to dysphagia and cough. One year ago, following the discovery of a mass in the right anterior cervical region, the patient was diagnosed with malignant thyroid cancer, squamous cell carcinoma, accompanied by extensive metastases to multiple sites including the trachea, larynx, medial segment of the right clavicle, sternum, bilateral submandibular lymph nodes, and pulmonary. Despite not undergoing thyroidectomy, the patient received comprehensive treatment including ablation and targeted therapy (no chemotherapy), with multiple hospitalizations during this period. The patient’s airway history included tracheal stent placement for dyspnea, later converted to tracheostomy due to stent migration. The tracheostomy was successfully closed after 1 month, though there was a 0.9 cm diameter stenosis 0.5 cm below the glottis. Preoperative neck imaging only revealed a 19 × 16 mm soft-tissue density mass occupying the supraglottic region of the anterior laryngeal wall, with subglottic airway narrowing observed 0.5 cm below the vocal cords (minimum lumen diameter: 9 mm); no TD was detected (Figure 1). Preoperative airway assessment revealed an inter-incisor distance of 5.5 cm (approximately three fingerbreadths) and Mallampati class II. Given this suspected difficult airway, ATI was planned for securing the airway prior to general anesthesia induction.

Upon operating room arrival, the patient’s vital signs were: heart rate (HR) 120 beats/min, blood pressure (BP) 150/80 mmHg, oxygen saturation (SpO₂) 99%, and respiratory rate (RR) 24 breaths/min. After preoxygenation via high-flow nasal cannula (FiO₂ 0.6, flow 50 L/min) and nebulization with 4 mL of 2% lidocaine (via “spray as you go”), two attempts at awake fiberoptic intubation were attempted, but neither visualized the glottis. On a third attempt by a senior anesthesiologist, the glottis was visualized but appeared edematous. After advancing past the glottis, tracheal rings were not visualized. We aborted the procedure, following multidisciplinary consultation, changed the surgical plan to nasogastric tube insertion. No other anesthetic drugs were added and the surgery was completed uneventfully in 20 min. Just prior to operating room discharge, the patient developed sudden drowsiness progressing to coma with vital signs: HR 140 beats/min, BP 97/46 mm Hg, SpO₂ 65%, and RR 5 breaths/min. Arterial blood gas revealed severe hypercapnia (PaCO₂ 102 mmHg). We immediately inserted a laryngeal mask airway (LMA) and initiated pressure-controlled ventilation (peak pressure 18 cm H₂O, PEEP 5 cm H₂O, RR 12/min). SpO₂ increased to 90% within 5 min. The patient regained consciousness and expelled the LMA spontaneously at 20 min (HR 95 beats/min, BP 100/53 mm Hg, SpO₂ 93%, and RR 15 breaths/min). Subsequently, a tracheotomy was performed at the site of the previous incision while the patient was conscious,and a 8.5#T-tube was placed immediately after performing tracheostomy. The patient was transferred to the intensive care unit for mechanical ventilation with vital signs as follows: HR 89 beats/min, BP 105/52 mm Hg, SpO₂ 95% and RR 18 breaths/min.

**Figure 2:**
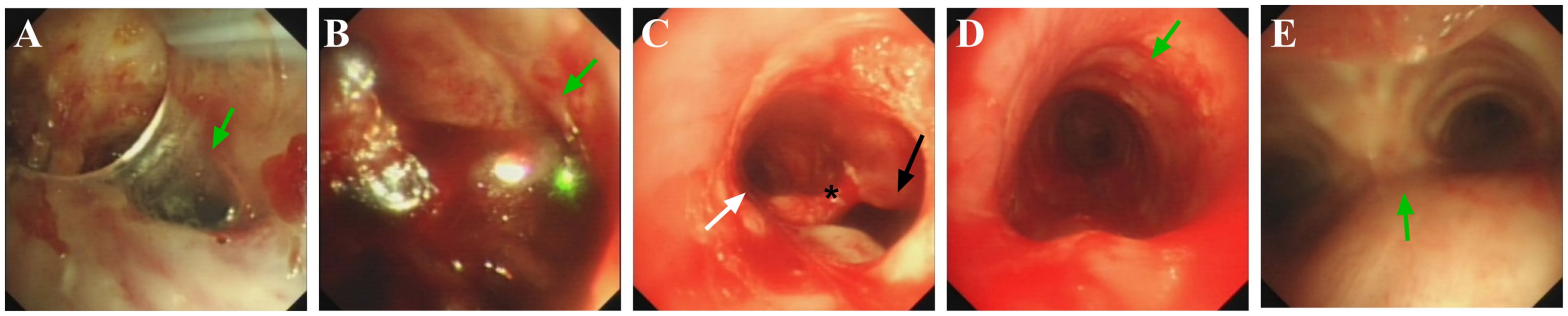
Postoperative airway fiberoptic bronchoscopy examination. Green arrow in **(A)**: the tracheostomy stoma. Green arrow in **(B)**: the absence of tracheal rings, with mucosal roughness and surface oozing. Arrows in **(C)**: the white arrow (left) is pointing toward the tracheal lumen; black arrow (right) is pointing toward the diverticulum opening; (*) marks the septum between the tracheal lumen and diverticulum. Green arrow in **(D)**: tracheal ring. Green arrow in **(D)**: carina.

After 20 h in the intensive care unit, the patient developed subcutaneous emphysema with audible crepitus at the incision site. Vital signs included HR 101 beats/min, BP 112/63 mmHg, SpO₂ 96%, RR 25 breaths/min, and End-tidal carbon dioxide (ETCO₂) 33 mmHg. Clinical examination confirmed an adequate seal between the T-tube and trachea. Bedside bronchoscopy revealed: (1) Inability to visualize tracheal rings distal to the tracheostomy site, with localized mucosal bleeding (Figures 2A,B); (2) A 0.5 cm subglottic stenosis immediately inferior to the vocal cords; (3) A diverticular orifice on the right posterolateral wall of the stenotic segment (Figure 2C), with a fistulous tract inferior to the opening; (4) Normal tracheal anatomy with identifiable tracheal rings and carina upon bronchoscope advancement (Figures 2D,E).

We removed the T-tube and positioned the patient supine. A 7.0 mm endotracheal tube (ETT) preloaded onto the FOB was advanced nasally into the trachea. Under direct vocal cord visualization, 5 mL of 2% lidocaine was administered through the bronchoscope’s working channel for topical anesthesia. Two symmetric luminal openings were identified approximately 0.5 cm below the glottis. The ETT was advanced 2 cm through the left orifice with visible tracheal rings. Transillumination of the lateral neck demonstrated no light transmission. ETCO₂ monitoring confirmed tracheal placement (approximately 35–45 mmHg). The tube was secured at 26 cm. On postoperative day 2, an otolaryngologist performed a bedside FOB-guided tracheostomy. The patient was placed in a supine position with a pillow under the shoulders. Inserted a FOB alongside the ETT to observe the main bronchi and removed any secretions from the airway. Under local infiltration anesthesia with 5 mL of 2% lidocaine administered at the anterior neck incision site, the operator stabilized the endotracheal tube while removing the sutures securing the inferior portion of the existing tracheostomy tract. A pointed scalpel was used to incise through the 2nd to 4th tracheal rings. After exposing the endotracheal tube, ventilation was paused and the tube was retracted to the subglottic region. Concurrently, the distal end of the FOB was withdrawn into the endotracheal tube lumen to prevent potential damage during subsequent maneuvers. Finally, an 8.5-mm single-use silicone tracheostomy tube was inserted under direct visualization with FOB guide. The ETT and FOB were subsequently withdrawn. A confirmatory bronchoscopy examination was then performed via the tracheostomy tube, verifying its optimal position within the tracheal lumen. The patient maintained stable vital signs throughout the procedure.

The patient was discharged on postoperative day 14. Written informed consent was obtained from the patient and her legally authorized representatives.

## 3 Discussion

For anticipated difficult airways, maintaining spontaneous ventilation remains paramount. ATI effectively prevents critical airway emergencies and is endorsed by the 2022 ASA guidelines as a first-line strategy (2). Supporting evidence includes a 12-year cohort study (2003–2013) of 146,252 patients (1,554 awake intubations) showing only a 2% failure rate for ATI (11). In this case, we attempted FOB-guided intubation, and after three unsuccessful attempts, we aborted the procedure. Failure to visualize tracheal rings during intubation typically indicates either esophageal intubation or entry into an abnormal anatomic structure. Rogers et al. (12) reported a 1:30 incidence of esophageal intubation in elective surgeries, while Russotto et al. (13) documented 5.6% (167/2959) in a multicenter study. Unrecognized esophageal intubation risks rapid hypoxemia, brain injury, and death (14). Since glottic passage was successful, we ruled out esophageal intubation, pointing to a tracheal anatomical abnormality. Subsequent bedside-FOB confirmed the diagnosis of a diverticulum.

**Figure 3:**
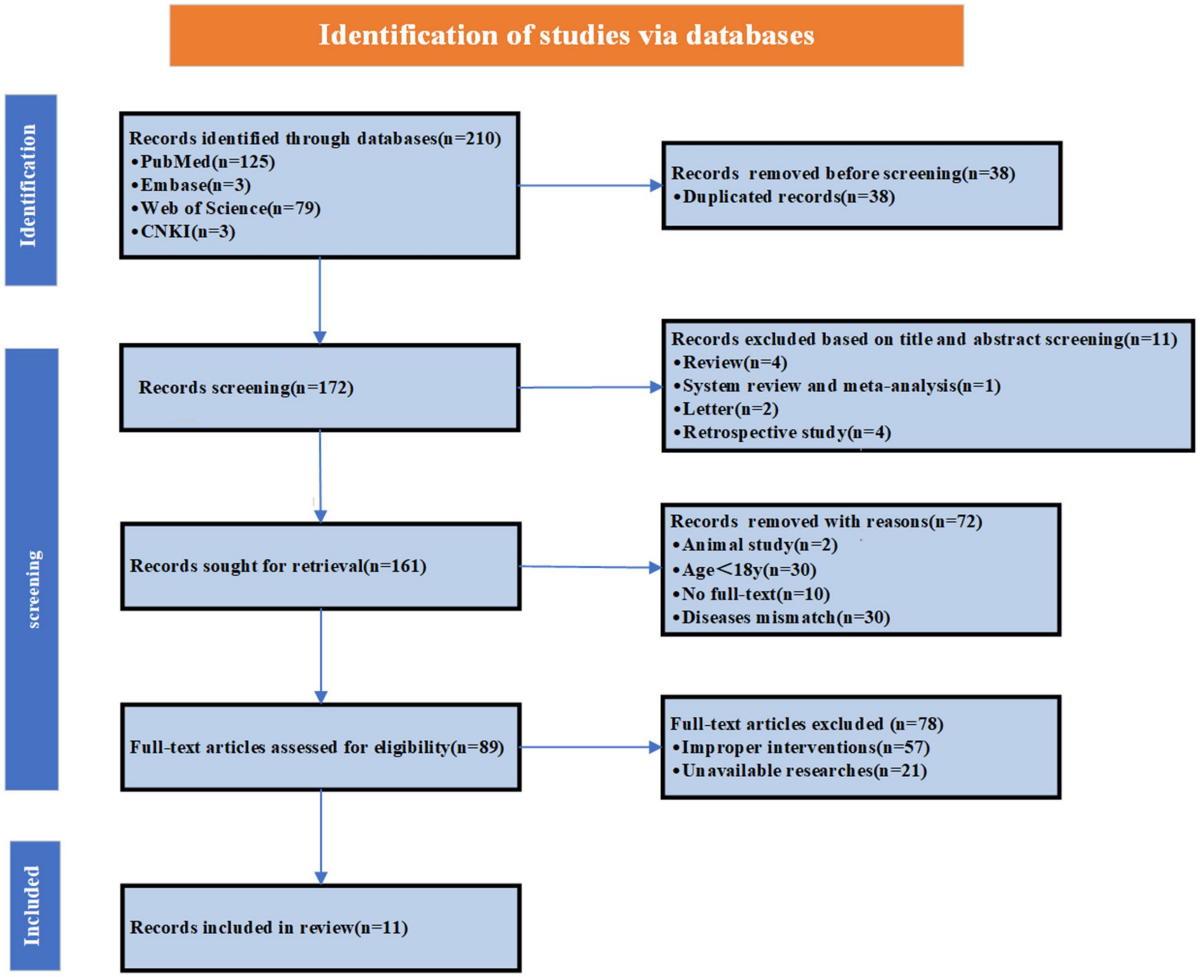
The flow diagram of the search strategy.

TD, a rare clinical entity, often escapes diagnosis due to nonspecific manifestations and low clinical suspicion. The first comprehensive clinicopathological description wasn’t published until 1954. This case highlights TD’s critical perioperative challenges. To improve anesthesiologists’ recognition and management of such patients, we systematically reviewed literature through September 25, 2025, searching PubMed, Web of Science, Embase, and China national knowledge infrastructure (CNKI) using “tracheal diverticul*.” We included English and non-English case reports of adults (≥18 years) with documented intubation details, excluding articles lacking intubation documentation (devices/attempts). Our search strategy (Supplementary Tables S1–S4; Figure 3) yielded 11 cases for analysis (Supplementary Table 5). The literature review demonstrated a preoperative TD detection rate of 18.2%, with 63.6% of patients asymptomatic and a mean age of 63.36 ± 14.1 years. Detailed data are presented in Table 1.

**Table 1:** Characteristics of the eligible cases for analysis.

Variable	Statistics
Age (years)	63.36 ± 14.14
Youngest	40
Oldest	80
40–59 years	4/11 (36.4%)
60–79 years	6/11 (54.5%)
≥80 years	1/11 (9.1%)
Sex	
Male	5/11 (45.5%)
Female	6/11 (54.5%)
Symptoms related to TD	
None	7/11 (63.6%)
Yes	4/11 (36.4%)
Preoperatively known	2/11 (18.2%)
First intubation tool: FOB	2/2 (100.0%)
1 attempt	2/2 (100.0%)
Airway related advert event	0/2 (0.0%)
Preoperatively unknown	9/11 (81.8%)
First intubation tool: laryngoscopic	9/9 (100%)
1 attempt	6/9 (66.7%)
2 attempts	2/9 (22.2%)
≥3 attempts	1/9 (11.1%)
Successful intubation technique: FOB	3/9 (33.3%)
Airway related advert event	9/9 (100%)
Diagnosed tool	
CT	8/11 (72.7%)
FOB	3/11 (27.3%)

TD, Tracheal diverticulum; FOB, Fiberoptic bronchoscopy; CT, Computed tomography.

Incidence increased with age, peaking in patients ≥60 years (*n* = 7, max age 80). This distribution aligns with the reported median onset age of 58 years (range, 16–93 years) (15). However, significant sex-based differences emerged: while Kurt et al. reported male predominance (64% vs. 36%) (15), and Marina Pace et al.’s CT study of 1,679 patients (124 TD cases) showed double the male incidence (16), our cohort demonstrated balanced distribution (45.5% vs. 54.5%). Anatomically smaller airway calibers and increased resistance in females may predispose them to greater diverticulum-related compromise under equivalent pathological stress, potentially explaining emerging female predominance in TD-related adverse events (17). Consistent with prior studies (15), 97.1% of diverticula occurred at the right posterolateral tracheal wall versus 2.9% left-sided. Among undiagnosed cases, 3 of 11 patients (27%) experienced ≥2 intubation failures with 100% complication rates. Multiple intubation attempts strongly correlate with complication incidence (18). In contrast, no complications occurred when preoperative airway assessment identified potential issues, featuring tailored preparation strategies and alternative intubation approaches. This discrepancy primarily stems from impaired subglottic visualization, where such lesions most commonly originate (15), a critical predictor of intubation failure. Consequently, preoperative assessment becomes essential for detecting occult airway pathologies. Imaging and endoscopic evaluation enable early detection of anatomical variants, thus guiding tailored intubation approaches to mitigate complications.

When TD is clinically suspected, imaging serves as the primary diagnostic tool. Computed tomography (CT), employed in 8 cases within this literature review, has become the preferred modality due to its capacity for comprehensive morphometric analysis, including quantification of dimensions, multiplicity, spatial topography, content composition, wall architecture, attachment integrity, and even differentiate between congenital and acquired cases. Unlike CT, X-ray fails to detect non-calcified cystic expansions and only identifies secondary complications like post-rupture pneumothorax, making it unsuitable for morphological characterization (9, 19, 20). However, CT carries significant false-negative rates, in Buterbaugh et al.’s series, only 9 of 26 (34.6%) TD were radiographically visible (21) This limitation chiefly stems from Valsalva maneuvers during CT acquisition: breath-holding elevates intrathoracic pressure, creating negative intraluminal gradients that cause diverticular collapse and non-visualization. While FOB allows direct luminal visualization and represents the diagnostic gold standard (22), its invasive nature limits routine preoperative use, reserving it primarily for emergent airway management. Major risk factors for suspected TD include: (1) chronic respiratory diseases, particularly alpha-1 antitrypsin deficiency, which increase tracheal wall pressure (23, 24); (2) prior surgical interventions such as tracheoesophageal fistula repair or tracheotomy (22, 25); (3) nonspecific symptoms including chronic cough, dyspnea, hoarseness, or dysphagia (3, 26). For high-risk patients, FOB should be recommended to detect potential mechanical obstructions from TD during airway instrumentation.

Per 2022 ASA guidelines, difficult airway management strategies must be tailored to the patient’s surgical context, clinical status, and the anesthesiologist’s expertise (2). A meta-analysis of 429 patients across 8 studies found no statistically significant difference in intubation failure or first-attempt success rates between video laryngoscopy and FOB for anticipated difficult intubation (27). Given the structural fragility of TD, FOB enables real-time subglottic navigation for precise visualization, proactively minimizing tissue trauma. Literature indicates one-third of undiagnosed diverticula cause initial intubation failure due to deceptive morphological resemblance between diverticular orifices and normal anatomy, increasing misidentification risk. Additionally, in patients with airway anatomical abnormalities, tracheal ring palpation alone may inadequately confirm ETT position, necessitating complementary techniques. In this case, ETT position was confirmed using tracheal ring palpation, assessment of light transmission through the incision, and capnography. Tracheal ring palpation is considered one of the “gold standards” for confirming ETT placement (28). This method, utilizing the FOB light source, may serve as an adjunct in specific scenarios, though its reliability can be influenced by operator experience and environmental factors (29). Capnography is a commonly used method to confirm ETT placement. According to 2022 ASA (2), capnography remains the preferred method for ETT position verification.

## 4 Conclusion

In summary, most data derived from published literature, resulting in a limited sample size that restricts generalizability. Potential selection bias and methodological constraints may further reduce clinical applicability. TD typically presents asymptomatically or with non-specific manifestations often obscured by primary comorbidities. For elective surgery patients with suspected TD, particularly those with prior neck surgery or disease, we advocate preoperative FOB to confirm anatomical defects and guide individualized airway management. This effectively reclassifies potential “unanticipated” difficult airways as “anticipated.” Our case validates transnasal FOB-guided intubation as an effective rescue strategy for unanticipated difficult airways during TD-related reintubation.

## Data Availability

The original contributions presented in the study are included in the article/Supplementary material, further inquiries can be directed to the corresponding author.

## References

[1] Sharma J Samagh N Kaur J Grewal A. Fiberoptic intubation in the lateral position in emergency airway Management of a Patient with large thyroid swelling. Turk J Emerg Med. (2025) 25:321–3. doi: 10.4103/tjem.tjem_206_24, 41104364 PMC12527047

[2] Apfelbaum JL Hagberg CA Connis RT Abdelmalak BB Agarkar M Dutton RP . 2022 American Society of Anesthesiologists Practice Guidelines for Management of the Difficult Airway. Anesthesiology. (2022) 136:31–81. doi: 10.1097/aln.0000000000004002, 34762729

[3] Chaudhry I Mutairi H Hassan E Afzal M Khurshid I. Tracheal diverticulum: a rare cause of hoarseness of the voice. Ann Thorac Surg. (2014) 97:e29–31. doi: 10.1016/j.athoracsur.2013.09.069, 24484838

[4] Mathey J Lemoine A. Tracheal diverticulum and congenital Oesophagotracheal fistula without Oesophageal atresia. Thorax. (1954) 9:106–11. doi: 10.1136/thx.9.2.106, 13179121 PMC1019354

[5] Tanrivermis Sayit A Elmali M Saglam D Celenk C. The diseases of airway-tracheal diverticulum: a review of the literature. J Thorac Dis. (2016) 8:E1163–7. doi: 10.21037/jtd.2016.10.92, 27867581 PMC5107528

[6] Early EK Bothwell MR. Congenital tracheal diverticulum. Otolaryngol Head Neck Surg. (2002) 127:119–21. doi: 10.1067/mhn.2002.126478, 12161741

[7] Frenkiel S Assimes IK Rosales JK. Congenital tracheal diverticulum. A case report. Ann Otol Rhinol Laryngol. (1980) 89:406–8. doi: 10.1177/000348948008900504, 6776862

[8] Goo JM Im JG Ahn JM Moon WK Chung JW Park JH . Right Paratracheal air cysts in the thoracic inlet: clinical and radiologic significance. AJR Am J Roentgenol. (1999) 173:65–70. doi: 10.2214/ajr.173.1.10397101, 10397101

[9] Soto-Hurtado EJ Peñuela-Ruíz L Rivera-Sánchez I Torres-Jiménez J. Tracheal diverticulum: a review of the literature. Lung. (2006) 184:303–7. doi: 10.1007/s00408-006-0010-7, 17086467

[10] Staffieri C Blandamura S Marioni G. Acquired tracheal diverticulum after tracheotomy. Am J Otolaryngol. (2013) 34:614–5. doi: 10.1016/j.amjoto.2013.03.002, 23578436

[11] Law JA Morris IR Brousseau PA De la Ronde S Milne AD. The incidence, success rate, and complications of awake tracheal intubation in 1,554 patients over 12 years: an historical cohort study. Can J Anaesth. (2015) 62:736–44. doi: 10.1007/s12630-015-0387-y, 25907462

[12] Rogers AM Hansel J Cook TM. Videolaryngoscopy, Oesophageal intubation and uncertainty: lessons from Cochrane. Anaesthesia. (2022) 77:1448–50. doi: 10.1111/anae.15818, 35897123

[13] Russotto V Myatra SN Laffey JG Tassistro E Antolini L Bauer P . Intubation practices and adverse Peri-intubation events in critically ill patients from 29 countries. JAMA. (2021) 325:1164–72. doi: 10.1001/jama.2021.1727, 33755076 PMC7988368

[14] Cook TM Nolan JP. Use of capnography to confirm correct tracheal intubation during cardiac arrest. Anaesthesia. (2011) 66:1183–4. doi: 10.1111/j.1365-2044.2011.06964.x, 22070599

[15] Kurt A Sayit AT Ipek A Tatar IG. A multi detector computed tomography survey of tracheal diverticulum. Eur J Med. (2013) 45:145–8. doi: 10.5152/eajm.2013.31, 25610271 PMC4261431

[16] Pace M Dapoto A Surace A Di Giacomo A Morzenti C Costantini E . Tracheal diverticula: a retrospective analysis of patients referred for thoracic Ct. Medicine. (2018) 97:e12544. doi: 10.1097/md.0000000000012544, 30278548 PMC6181548

[17] Bhatt SP Bodduluri S Nakhmani A Kim YI Reinhardt JM Hoffman EA . Sex differences in Airways at Chest Ct: results from the Copdgene cohort. Radiology. (2022) 305:699–708. doi: 10.1148/radiol.212985, 35916677 PMC9713451

[18] Mort TC. Emergency tracheal intubation: complications associated with repeated laryngoscopic attempts. Anesth Analg. (2004) 99:607–13. doi: 10.1213/01.Ane.0000122825.04923.15 15271750

[19] Yagyu T Saito H Kono Y Murakami Y Kuroda H Matsunaga T . Thoracic esophagus Cancer revealing a tracheal diverticulum. Yonago Acta Med. (2017) 60:200–3. doi: 10.33160/yam.2017.09.010, 28959132 PMC5611476

[20] Rojas J Bostanci K Katsenos S Becker HD. Tracheal diverticulum. J Bronchol Interv Pulmonol. (2011) 18:91–3. doi: 10.1097/LBR.0b013e318205990b, 23169027

[21] Buterbaugh JE Erly WK. Paratracheal air cysts: a common finding on routine Ct examinations of the cervical spine and neck that may mimic pneumomediastinum in patients with traumatic injuries. AJNR Am J Neuroradiol. (2008) 29:1218–21. doi: 10.3174/ajnr.A1058, 18544671 PMC8118841

[22] Zhou W Lang Y Xu Z Yin D. Rare and unexpected ventilation difficulties due to tracheal diverticulum: a case report. Medicine. (2023) 102:e34536. doi: 10.1097/md.0000000000034536, 37565856 PMC10419496

[23] Amaral CB Silva S Feijó S. Infected tracheal diverticulum: a rare association with Alpha-1 antitrypsin deficiency. J Bras Pneumol. (2014) 40:669–72. doi: 10.1590/s1806-37132014000600011, 25610508 PMC4301252

[24] Koudounarakis E Karatzanis A Velegrakis S Fountoulakis E Velegrakis G. A case of acquired tracheal diverticulum presenting with Globus Pharyngeus. Am J Otolaryngol. (2013) 34:82–4. doi: 10.1016/j.amjoto.2012.08.013, 23084429

[25] Cheng AT Gazali N. Acquired tracheal diverticulum following repair of Tracheo-Oesophageal fistula: endoscopic management. Int J Pediatr Otorhinolaryngol. (2008) 72:1269–74. doi: 10.1016/j.ijporl.2008.04.002, 18524390

[26] Ceulemans LJ Lerut P De Moor S Schildermans R De Leyn P. Recurrent laryngeal nerve paralysis by compression from a tracheal diverticulum. Ann Thorac Surg. (2014) 97:1068–71. doi: 10.1016/j.athoracsur.2013.06.118, 24580928

[27] Alhomary M Ramadan E Curran E Walsh SR. Videolaryngoscopy vs. fibreoptic bronchoscopy for awake tracheal intubation: a systematic review and meta-analysis. Anaesthesia. (2018) 73:1151–61. doi: 10.1111/anae.14299, 29687891

[28] Salem MR. Verification of endotracheal tube position. Anesthesiol Clin North Am. (2001) 19:813–39. doi: 10.1016/s0889-8537(01)80012-2, 11778382

[29] Knapp S Kofler J Stoiser B Thalhammer F Burgmann H Posch M . The assessment of four different methods to verify tracheal tube placement in the critical care setting. Anesth Analg. (1999) 88:766–70. doi: 10.1097/00000539-199904000-00016, 10195521

