# Supplementary material for: Case Report: Emergency management of difficult airway in a thyroid cancer patient with undiagnosed tracheal diverticulum preoperatively and literature review
Source: Front Med (Lausanne). 2026 Jan 2;12:1739525. doi: 10.3389/fmed.2025.1739525 (PMC12808488; doi:10.3389/fmed.2025.1739525)
Supplement: Supplementary file 1 [file Table_1.DOCX]

Supplementary Table 1. Search strategy in PubMed.

PubMed (Performed on September 24th, 2025)

| Number | Searched for |
| --- | --- |
| #1 | “tracheal diverticulum disease”[MeSH Terms] |
| #2 | “tracheal diverticulum*”[Title/Abstract] |
| #3 | “tracheal diverticulosis*”[Title/Abstract] |
| #4 | #1 OR #2 OR #3 |
| #5 | “Case Reports”[Publication Type] |
| #6 | “Case Study”[Title/Abstract] |
| #7 | “Case Studies”[Title/Abstract] |
| #8 | “Case Histories”[Title/Abstract] |
| #9 | #5 OR #6 OR #7 OR #8 |
| #10 | #9 NOT (animals[MeSH Terms] NOT (humans[MeSH Terms])) |
| #11 | #4 AND #10 |
