# Supplementary material for: Case Report: Emergency management of difficult airway in a thyroid cancer patient with undiagnosed tracheal diverticulum preoperatively and literature review
Source: Front Med (Lausanne). 2026 Jan 2;12:1739525. doi: 10.3389/fmed.2025.1739525 (PMC12808488; doi:10.3389/fmed.2025.1739525)
Supplement: Supplementary file 2 [file Table_2.DOCX]

Supplementary Table 2. Search strategy in Embase.

Embase(Performed on September 24th, 2025)

| Number | Searched for |
| --- | --- |
| #1 | 'tracheal diverticulum':ti,ab,kw |
| #2 | 'tracheal diverticulosis':ti,ab,kw |
| #3 | #1 OR #2 |
| #4 | 'case reports as topic'/exp |
| #5 | 'case study':ti,ab,kw |
| #6 | 'case studies':ti,ab,kw |
| #7 | 'case histories':ti,ab,kw |
| #8 | #4 OR #5 OR #6 OR #7 |
| #9 | #3 AND #8 |
