# Supplementary material for: Case Report: Emergency management of difficult airway in a thyroid cancer patient with undiagnosed tracheal diverticulum preoperatively and literature review
Source: Front Med (Lausanne). 2026 Jan 2;12:1739525. doi: 10.3389/fmed.2025.1739525 (PMC12808488; doi:10.3389/fmed.2025.1739525)
Supplement: Supplementary file 3 [file Table_3.DOCX]

Supplementary Table 3. Search strategy in Web of Science.

Web of Science Core Collection (Performed on September 24th, 2025)

| Number | Searched for |
| --- | --- |
| #1 | TS=(tracheal diverticulum disease OR tracheal diverticulum* OR tracheal diverticulosis* ) |
| #2 | TS=(Case Reports OR Case Study OR Case Studies OR Case Histories) |
| #3 | TS=(animals) |
| #4 | TS=(humans) |
| #5 | (#3) NOT #4 |
| #6 | (#2) NOT #5 |
| #7 | (#1) AND #6 |
