# Supplementary material for: Case Report: Emergency management of difficult airway in a thyroid cancer patient with undiagnosed tracheal diverticulum preoperatively and literature review
Source: Front Med (Lausanne). 2026 Jan 2;12:1739525. doi: 10.3389/fmed.2025.1739525 (PMC12808488; doi:10.3389/fmed.2025.1739525)
Supplement: Supplementary file 4 [file Table_4.DOCX]

Supplementary Table 4. Search strategy in CNKI.

China National Knowledge Infrastructure (CNKI) (Performed on September 24th, 2025)

| Number | Searched for |
| --- | --- |
| #1 | TKA=气管憩室 |
| #2 | TKA=气管憩室病 |
| #3 | #1 OR #2 |
| #4 | SU=病例报告 |
| #5 | TKA=个案研究 |
| #6 | TKA=病案史 |
| #7 | TKA=病例研究 |
| #8 | #3 OR #4 OR #5 OR #6 OR #7 |
| #9 | #3 AND #8 |
